# Multiple phenotypic changes in mice after knockout of the *B3gnt5 *gene, encoding Lc3 synthase--a key enzyme in lacto-neolacto ganglioside synthesis

**DOI:** 10.1186/1471-213X-10-114

**Published:** 2010-11-18

**Authors:** Chien-Tsun Kuan, Jinli Chang, Jan-Eric Mansson, Jianjun Li, Charles Pegram, Pam Fredman, Roger E McLendon, Darell D Bigner

**Affiliations:** 1 Department of Pathology and the Preston Robert Tisch Brain Tumor Center at Duke, Duke University Medical Center, Durham, NC 27710 USA; 2 Department of Psychiatry and Neurochemistry, Institute of Neuroscience and Physiology, The Sahlgren Academy at University of Gothenburg, Sahlgren University Hospital/Molndal, SE-431 80 Molndal, Sweden

## Abstract

**Background:**

Ganglioside biosynthesis occurs through a multi-enzymatic pathway which at the lactosylceramide step is branched into several biosynthetic series. Lc3 synthase utilizes a variety of galactose-terminated glycolipids as acceptors by establishing a glycosidic bond in the beta-1,3-linkage to GlcNaAc to extend the lacto- and neolacto-series gangliosides. In order to examine the lacto-series ganglioside functions in mice, we used gene knockout technology to generate Lc3 synthase gene *B3gnt5-*deficient mice by two different strategies and compared the phenotypes of the two null mouse groups with each other and with their wild-type counterparts.

**Results:**

*B3gnt5 *gene knockout mutant mice appeared normal in the embryonic stage and, if they survived delivery, remained normal during early life. However, about 9% developed early-stage growth retardation, 11% died postnatally in less than 2 months, and adults tended to die in 5-15 months, demonstrating splenomegaly and notably enlarged lymph nodes. Without lacto-neolacto series gangliosides, both homozygous and heterozygous mice gradually displayed fur loss or obesity, and breeding mice demonstrated reproductive defects. Furthermore, *B3gnt5 *gene knockout disrupted the functional integrity of B cells, as manifested by a decrease in B-cell numbers in the spleen, germinal center disappearance, and less efficiency to proliferate in hybridoma fusion.

**Conclusions:**

These novel results demonstrate unequivocally that lacto-neolacto series gangliosides are essential to multiple physiological functions, especially the control of reproductive output, and spleen B-cell abnormality. We also report the generation of anti-IgG response against the lacto-series gangliosides 3'-isoLM1 and 3',6'-isoLD1.

## Background

Gangliosides constitute a large group of sialylated glycosphingolipids (GSLs), which are preferentially (the concentrations intracellularly are most likely higher) expressed on the outer leaf of plasma membranes. The clusters of most negatively charged gangliosides are associated mainly with membranes of either hematopoietic progenitors or stromal cells of a variety of tissues. Functionally, gangliosides influence cell growth and death, probably because they are involved in the glyco-mediated assembling of signaling molecules, such as growth factor receptors or integrins, and cell adhesion molecules and their ligands [1-4], which further modulate the signaling pathway [5,6]. Gangliosides help to determine the microenvironment inside a cell [7]--its physical or chemical properties, local pH, calcium homeostasis, etc. [8], which could enhance or abrogate the biological availability of signaling molecules and disrupt their interactions. All of these conditions within the cell influence its ability to regulate cell proliferation and differentiation and cell-cell contact, as well as oncogenesis and hematopoiesis. Several lines of research show that gangliosides serve not only as functional molecules for cell development and growth, but also as biological markers for cell sorting or as potential targets in tumor therapy, because aberrant ganglioside expression has been known to occur in many cancers, such as lymphoma, neuroblastoma, glioma, melanoma, breast cancer, and small cell lung carcinoma [9-13].

**Figure 1:**
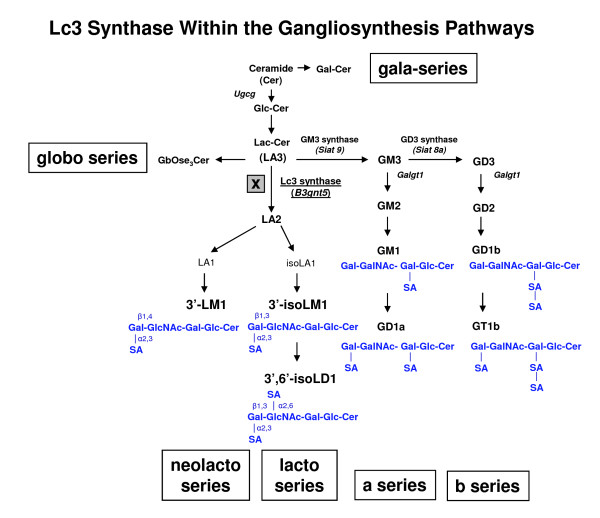
**Lc3 synthase within the gangliosynthesis pathways**. The X in the lacto-neolacto series biosynthesis pathway indicates a block due to disruption of the Lc3 synthase gene, *B3gnt5*. Six ganglioside synthesis pathways are shown: globo, gala, neolacto, lacto, a, and b. The molecules 3'-LM1, 3'-isoLM1, and 3',6'-isoLD1 are shown within the lacto-neolacto pathways. Gangliosides GM1 and GD1a are shown in the a-series pathway and ganglioside GD1b in the b-series pathway. Ganglioside nomenclature according to Svennerholm [44].

Gangliosides are distinguished by the actions of specific core glycosyltransferases (Figure 1). The specific roles of gangliosides remain incompletely understood. Nonetheless, because of the dramatic change in their expression during neuronal developmental differentiation and brain morphogenesis, as well as their prominence in the mature central nervous system (CNS), gangliosides are assumed to have fundamental roles in the CNS [8,10].

Specific core glycosyltransferase gene knockout in mice has proven to be a particularly useful approach for uncovering the functions of gangliosides in the brain [1]. In 2003, for example, Yamashita et al. knocked out GM3 synthase (*Siat9 *gene, CMP-NeuAc: lactosylceramide α-2,3-sialyltransferase, EC 2.4.99.9) in the a-series pathway [14]. Mice that carried the mutation of GM3 synthase remained normal as compared to the wild type. In the b series, mice with disruption of GD3 synthase (*Sia8a *gene, CMP-sialic acid: GM3 a-2,8-sialyltransferase, EC 2.4.99.8) showed a relatively normal phenotype [15]. When GM2/GD2 synthase (*Galgt1 *gene, UDP-*N*-acetyl-D-galactosamine: GM3/GM2/GD2 synthase, also known as *GalNAcT*, EC 2.4.1.92) was disrupted [16], the mutant mice had no overt abnormalities in appearance and experienced a nearly normal life span. However, they did show evidence of dysmyelination and some axonal degeneration. The reasons for the normal life of these single gene knockout mice are not clear. It may be that there is functional overlap such that only a partial disruption of ganglioside synthesis does not elicit severe pathology. Thus, double knockout mice have been generated for further elucidation of ganglioside functions. In contrast to the relatively subtle phenotypic changes of mutant mice with a single deletion of the glycosyltransferase gene, double knockout of the *Galgt1 *and *Sia8a *genes displayed a sudden-death phenotype and a severe CNS disturbance by lethal, sound-induced seizures [15]. Likewise, by genetic engineering of both the *Galgt1 *and the *Siat9 *genes, the mutant mice developed a severe neurodegenerative disease that resulted in death [17]. The results with DKO mice thus indicated that both the a-series and the b-series gangliosides play a pivotal role in stabilizing the CNS. Moreover, when the *GlcCer synthase *gene (Ugcg: UDP-glucose:ceramide glucosyltransferase, EC2.4.1.80) was knocked out, which disrupted the initial step in most ganglioside biosynthesis pathways, the mice died at embryonic days 6.5 to 7.5 (days E6.5-E7.5) [18]. Knocking out this gene in the brain produced severe neural defects and abnormalities in neural cell differentiation and caused death of all mice within 24 days, which further underscores the physiological importance of gangliosides in the CNS [19,20].

We have previously demonstrated that several structures in the lacto-neolacto-series pathways, such as the gangliosides 3'-isoLM1 and 3',6'-isoLD1, were overexpressed in brain tumors [11]. However, the biological role of these gangliosides has not been fully elucidated. The aim of this study was to establish a knockout mouse model lacking the lactoseries GSLs and to investigate the biological significance of Lc3 synthase, which initiates the formation of lacto-neolacto-chain gangliosides (Figure 1). In our previous attempts to generate monoclonal antibodies (MAbs) to target the glioma-specific gangliosides 3'-isoLM1 and 3',6'-isoLD1, we immunized mice with these two gangliosides to develop antibodies against them. However, we isolated only 3'-isoLM1- and/or 3',6'-isoLD1-specific IgM antibodies, which are unsuitable for therapy, rather than IgG antibodies, which are more suitable for therapeutic use. In the past, it has been difficult to produce high-affinity, IgG MAbs to glycolipid and ganglioside antigens because of the prevalence of a primitive, low-affinity IgM response by most mammals to these usually highly conserved structures. However, Proia's group [21] has recently developed transgenic knockout mice in which the GM2/GD2 synthase enzyme gene (*Gal-NAcT*) is knocked out. Such mice have then been used to prepare high-affinity IgG antibodies against complex gangliosides in the ganglioside series [22]. Similarly, we knocked out the Lc3 synthase gene *B3gnt5*, and "immunologically naïve" mice lacking lacto-neolacto-series gangliosides were thus generated for immunization purposes to produce high-affinity IgG MAbs against the tumor-associated gangliosides 3'-isoLM1 and 3',6'-isoLD1.

Lc3 synthase is a member of the beta-1,3-*N*-glycosyltransferase superfamily, coded by the *B3gnt5 *gene on chromosome 16 B1 of the mouse genome. The mouse cDNA of Lc3 synthase includes an opening reading frame of 1131 base pairs encoding a protein of 376 amino acids, a type II membrane protein. Lc3 synthase exhibits strong activity in transferring GlcNAc to the lactosylceramide for lacto-neolacto-chain ganglioside biosynthesis (Figure 1), and this activity is regulated during embryonic development, especially during brain morphogenesis. In adults, Lc3 synthase is expressed mainly in spleen, placenta, and cerebellum [23].

In our study, the null mouse pups, in which the Lc3 synthase gene was disrupted, showed no obvious phenotypic changes if they survived delivery. However, 11% died in less than two months, and about 9% of the surviving offspring developed growth retardation. Over time, alterations in multiple organs were observed. The obvious phenotypic changes were not in the CNS, as in the a- and b-series ganglioside mutant mice, but in reproductive output and spleen B-cell abnormality. Here, for the first time, we report those phenotypic changes from null lacto-neolacto-series knockout mice, as well as the generation of an anti-IgG response against the lacto-series gangliosides 3'-isoLM1 and 3',6'-isoLD1.

## Results

### Establishment of the knockout model in mice

**Figure 2:**
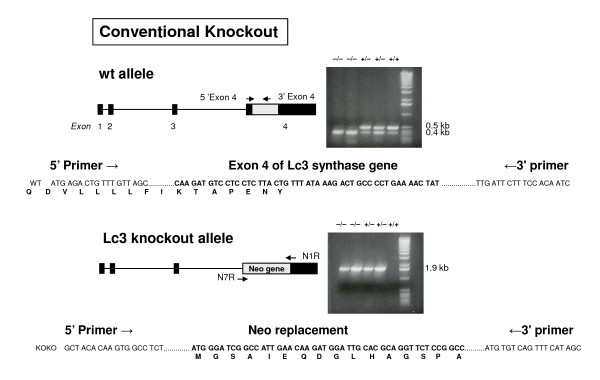
***B3gnt5 *gene knockout, conventional method**. Conventional method for targeted disruption of *B3gnt5*. Given the genomic structure of the mutant allele, we designed primers spanning exon 4 and inserted a *neo *gene to distinguish all three genotypes. With exon 4 primers, knockout mice (-/-) displayed a nonspecific band at about 0.4 kb, as shown in lanes 1 and 2 of the PCR gel, with sequencing confirmation of nonspecific product. For heterozygous (+/-) and wild-type (+/+) mice, two bands were viewed at 0.4 kb and 0.5 kb. Sequence analysis of the 0.5-kb band confirmed that it was exon 4 of *B3gnt5*. With the inserted *neo *gene primer pair (N1R, N7R), both homozygous (-/-) and heterozygous (+/-) mice carried a positive 1.9-kb band, whereas a negative result in this PCR analysis indicated wild type (+/+).

**Figure 3:**
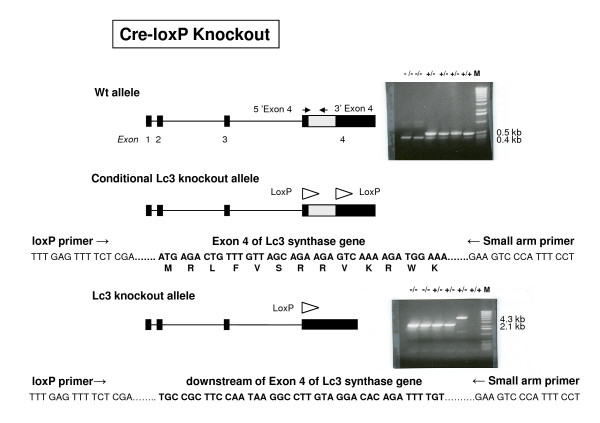
***B3gnt5 *gene knockout, Cre-loxP method**. Cre-loxP method for targeted disruption of *B3gnt5*. For the Cre-loxP model, two primer pairs were used also. The exon 4 primer pair was the same as that used for conventional knockout (Figure 2). Results were analyzed as for the conventional knockout method. Another primer set detected the inserted loxP site. Both homozygous (-/-) and heterozygous (+/-) mice carried a positive 2.1-kb band, which resulted from exon 4 deletion by Cre recombinase. A 4.3-kb band indicated when the targeted portion was not deleted by Cre recombinase. No band appeared in this PCR analysis for the wild-type mice (+/+). The PCR products were sequenced to further confirm the homogeneity.

**Figure 4:**
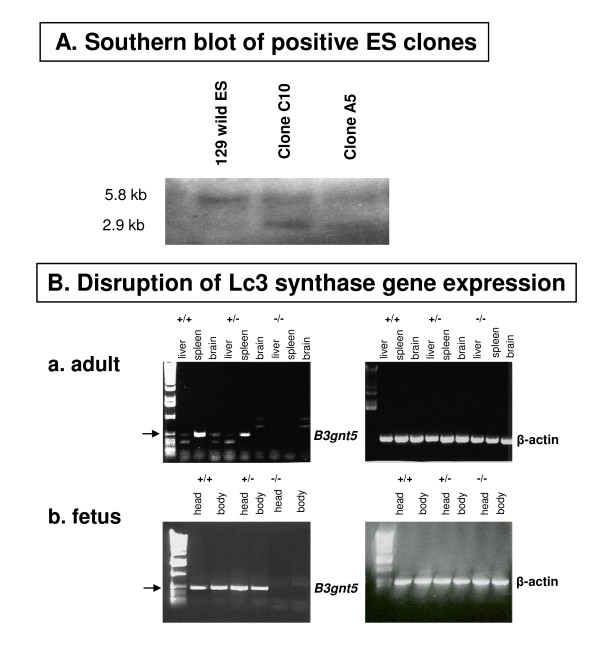
***B3gnt5 *gene knockout analysis**. **A**. Genomic Southern blot of ES cell clones. Genomic DNA from wild-type ES clones and possible candidate ES clones carrying homologously recombinant *B3gnt5 *allele were digested with the Xba I restriction enzyme and hybridized to the genomic probe. Two bands are shown, a 5.8-kb band of wild type and a 2.9-kb band (from Clones C10 and A5), indicating that the recombination occurred. **B**. Disruption of Lc3 synthase gene expression shown by RT-PCR. Disruption of Lc3 synthase gene expression shown by RT-PCR for the wild type (+/+), heterozygote (+/-), and homozygote (-/-). Total RNA was extracted from different organs in both adult and fetus and reversely transcripted into cDNA. PCR was performed by using cDNA as a template and primers located in exon 4 of Lc3 synthase. Beta actin was the internal quality control (right panels). **a**. Results from three different tissues in adult mice. In the wild-type (+/+) genotype, Lc3 synthase gene expression was detected mainly in spleen and was weakly positive in brain and liver. Lc3 synthase expression was decreased in the heterozygote (+/-) and completely knocked out in the homozygote (-/-). **b**. Results from head and body of E17 fetuses. Lc3 synthase gene expression was detected in both head and body tissue in the wild-type (+/+) fetus and the heterozygote (+/-) but was not detected in either head or body of the homozygote (-/-).

The lacto-neolacto-series ganglioside pathways should be completely disrupted by *B3gnt5 *gene knockout (Figure 1). We established two knockout models, one by a conventional method (Figure 2) and one with a Cre-loxP system (Figure 3). The models differ in two major features. For the conventional method, coding region exon 4 and part of intron 3 were knocked out and replaced by the *neo *gene cassette, with the progenitor background of C57BL/6, whereas only exon 4 was knocked out by the Cre enzyme for the Cre-loxP model, with the chimerical background of 129sv and C57BL/6 in the offspring. The targeted embryonic stem (ES) cells were screened and confirmed by Southern blot analysis (Figure 4A), and the correct targeting and deletion of the Lc3 synthase allele was then confirmed by genotyping PCR and PCR product sequencing (Figure 2, 3).

The complete absence of Lc3 synthase transcripts from several tissues of null mice was further demonstrated by RT-PCR in both adult and fetus (Figure 4B, panels a and b, respectively), in contrast to tissue results for the Lc3 synthase heterozygote and wild type. In the wild-type adult, Lc3 synthase was strongly expressed in spleen, while weakly expressed in liver and brain (Figure 4B.a). Heterozygotes expressed less Lc3 synthase in spleen, which is consistent with one copy of the gene being knocked out. In an E17 fetus, both head and body showed strong expression of Lc3 synthase in wild-type (Wt/Wt) and heterozygous (Wt/KO) mice (+/+ and +/-, respectively, in Figure 4B.b). However, tissue from the Lc3 synthase homozygote, whether it was an adult or a fetus, showed no transcripts at all (Figure 4B).

To explore whether there was any residual activity of Lc3 synthase in knockout mice, we quantitated the lacto-neolacto ganglioside series (Table 1). 3'-LM1 and 3'-isoLM1 are the major downstream components in the lacto-neolacto-series pathways (Figure 1). The total ganglioside content (expressed as μmol sialic acid per gram of tissue) and the distribution of the major gangliotetraose gangliosides GM1, GD1a, GD1b, and GT1b, which constitute about 90% of the total ganglioside sialic acid content, were used as internal positive controls to exclude any major effect on ganglioside topography. There were no significant differences among the three genotypes, KO/KO, Wt/KO, and Wt/Wt, either in the total ganglioside concentration or in the distribution of the major ganglioteraose-series gangliosides (GM1, GD1a, GD1b, and GT1b), in cerebellum or cortex tissue (Table 1). This indicated that the ganglioside biosynthesis in the a- and b-series was not affected by the *B3gnt5 *gene knockout. The results from adults showed that the cerebellum expressed a detectable amount of the neolacto-series ganglioside 3'-LM1, about 0.24 nmol/g of tissue for wild-type mice, and Lc3 synthase heterozygous knockout mice expressed about half that amount, 0.14 nmol/g of tissue. In contrast, the Lc3 synthase homozygous knockout mice were totally lacking in 3'-LM1 expression. The results from cortex revealed that only the Lc3 synthase wild-type mice expressed 3'-LM1, about 0.18 nmol/g of tissue. Notably, the wild-type fetus showed 10-35-fold higher expression of 3'-LM1 than did the adult, even in comparing results for whole brain in the fetal assay against results for cerebellum in the adult assay. This is consistent with RT-PCR results, indicating that the wild-type fetus contains more Lc3 synthase. Conversely, we detected no 3'-isoLM1 in either wild-type adult or fetus (Table 1), possibly because 3'-isoLM1 is a very minor component of the total series of gangliosides and, with its relatively low concentration, might not be detectable by our method.

**Table 1:** Ganglioside concentration of Lc3 synthase knockout mice*

**Tissue and Genome Type**^†^	Sialic Acid (μmol/g tissue)	GM1 %SA	GD1a %SA	GD1b %SA	GT1b %SA	3'-LM1 (nmol/g tissue)	3'-isoLM1 (nmol/g tissue)
**Adult Cerebellum**							
KO/KO	2.64 ± 0.33	7.45 ± 1.50	8.78 ± 1.23	18.9 0 ± 1.17	47.6 ± 1.69	ND	ND
Wt/KO	2.70 ± 0.28	7.00 ± 1.90	7.63 ± 0.75	18.58 ± 1.71	51.8 ± 3.14	0.14 ± 0.12	ND
Wt/Wt	2.64 ± 0.25	6.15 ± 0.85	7.68 ± 0.81	19.13 ± 1.74	51.3 ± 2.46	0.24 ± 0.13	ND
**Adult Cortex**							
KO/KO	3.85 ± 0.38	8.35 ± 1.06	33.7 ± 4.14	8.90 ± 0.49	38.7 ± 1.83	ND	ND
Wt/KO	3.95 ± 0.66	8.38 ± 1.42	37.3 ± 4.35	8.58 ± 1.04	38.0 ± 3.08	ND	ND
Wt/Wt	3.73 ± 0.37	7.35 ± 0.55	34.7 ± 3.71	8.90 ± 0.42	40.2 ± 2.04	0.18^§^	ND
**Fetus brain**							
KO/KO						ND	ND
Wt/KO						1.40 ± 0.00	ND
Wt/Wt						5.00 ± 0.42	ND

KO, knockout; ND, not detected; Wt, wild type; %SA, percentage of sialic acid.

*All values are means ± SD. The lacto-series ganglioside assay was based on lipid extracts corresponding to 5 mg of tissue that were assayed by TLC-immunostaining. Detection limits were as follows: 3'-LM1, 0.04 nmol/g tissue; 3'-isoLM1, 0.04 nmol/g tissue.

^†^Four samples were in each group for the cerebellum assay and the cortex assay. Two samples were in each group from different parental pairs for the fetal brain assay.

^**#**^Remaining gangliosides constituting 100% of total sialic acid: GM3, GM2, GD3, GD2, and GQ1.

^§^Data from one mouse detected.

### Phenotypic changes of Lc3 synthase knockout mice

#### Early stage growth delay

**Figure 5:**
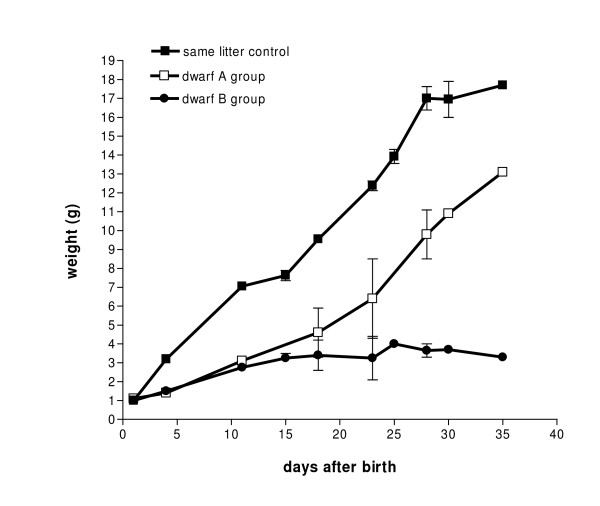
**Early-stage growth delay of dwarfism in mice following Lc3 synthase knockout**. The breeding was set in a heterozygote-heterozygote (+/-, +/-) base. Pups were weighed periodically. Mice of normal weight were weaned at day 21, and mice weighing <10 g were kept in the parental cage. The average weight of the same-litter control differs significantly from that of the dwarf B group (*P *= 0.0008), but differs less from that of the dwarf A group (*P *= 0.1072). Composition of the three mouse groups: Same-litter control (■): one +/+, two -/+, and one -/-; dwarf A group (□): two -/+; dwarf B group (●): two -/+.

Once the knockout models were established, we maintained colonies through cross-breeding of heterozygotes. Of the pups that survived 5 days, about 80% showed normal postnatal development, in terms of suckling, righting, and eye opening. However, about 9% of the pups showed growth delay, or dwarfism, especially in the first 20 postnatal days. Genotyping revealed that, in the total of 12 mice with the dwarf phenotype, only 2 were homozygous and 2 wild type, and 8 were heterozygous. Additionally, we completed a genotype analysis of all five pups in one particular litter, which included one dwarf that was heterozygous, and four littermates that displayed normal growth rate and represented all three genotypes, indicating that the dwarf phenotype may not be directly related to knockout of the Lc3 synthase gene *B3gnt5*. This group of 4 littermates served as the same-litter control that is represented in Figure 5. We noted that the delayed-growth group could be further divided into two patterns: One pattern showed growth delay in the early stage after birth, within about 20 days, and then the growth in these pups gradually caught up to that of the normal mice (Figure 5, dwarf group A); the other pattern showed growth arrest at about 10 days, and these pups then died some time after that (dwarf group B).

#### Lower survival rate after birth

The genotype distribution studies on all offspring identified by PCR showed that, for fetuses from both the conventional and the Cre-loxP models, heterozygous breeding produced offspring at the expected Mendelian frequency, 14%-25% for wild type, 29%-32% for homozygote, and 43%-57% for heterozygote (Table 2). However, in mature offspring, the survival rate for homozygous mice showed a 50% drop--11%-16% (mature offspring) versus 29%-32% (fetus), whereas the survival rate for wild-type mice increased from 14%-25% (fetus) to 32%-50% (mature offspring). One notable feature for the Cre-loxP model was that in the total 251 surviving pups, 50% were of the wild type, which was twofold higher than the expected 25% Mendelian frequency (*P *< 0.0001). This phenomenon became more pronounced in mice that died at the age of 2 months. About 71% of these dead mice were homozygotes with no wild type, and they displayed no symptoms, which indicates that Lc3 synthase homozygous knockout mice were more predisposed to death in early life (KO/KO genome in Table 2). We suspect that the deaths were due to early-stage developmental defects.

**Table 2:** Genome typing distribution in three groups of Lc3 synthase knockout mice*

Group	Number of mice	Wt/Wt (% of total)	Wt/KO (% of total)	KO/KO (% of total)
Fetus^†^				
Conventional	28	7 (25)	12 (43)	9 (32)
Cre-loxP	14	2(14)	8 (57)	4 (29)
Adult^§^				
Conventional	127	40 (32)	66 (52)	21 (16)**
Cre-loxP	251	126 (50)^§§^	97 (39)^‡‡^	28 (11)^§§^
Dead^§^				
Conventional	14	0**	4 (29)^‡‡^	10 (71)^§§^

Abbreviations: KO, knockout; Wt, wild type.

*Genome typing was done at day E17 for fetuses, after weaning for adult mice, and immediately after death in postnatal days 15-40.

^†^Fetuses were obtained through dissection of a dam as described in Materials and Methods.

^§^Values and percentages in the Adult and Dead groups (Wt/Wt, Wt/KO, and KO/KO) were significantly different from the theoretical number based on Mendel's law.

**P < 0.05.

^§§^*P *< 0.00001 by binomial test.

^‡‡^*P *< 0.0004.

In the heterozygous-heterozygous breeding, we found that the survival rate of pups after birth was significantly lower than that from normal mouse breeding. The survival of offspring from conventional knockout mice dramatically decreased, to a rate of about 59%, versus the expected 100% survival rate from normal mouse breeding, with a *P *value of < 0.0001 (Table 3A). Most of those pups died during delivery or immediately after birth. Although the Cre-loxP model showed a slightly higher survival rate--about 89%, statistical analysis still revealed a significant difference versus the expected 100% survival rate from normal mouse breeding, also with a *P *value of < 0.0001.

**Table 3:** Survival rate and reproductive effects of Lc3 synthase knockout: Heterozygote breeding analysis in the further study*^†^

Outcome of Breeding	Conventional Knockout	Cre-loxP Knockout
Number of delivered offspring	96	16
Number of surviving mice (survival rate)	41 (43%)^§^	9 (56%)^†^
Number of breeding pairs	19	13
Breeding time (months)	5	5
Number of litters	26	10
Average litter #/breeding pair	1.4	1.3
Average # of pups/litter (av # surviving/litter)	3.7 (1.6)	1.6 (0.9)

*The breeding was carried out with a heterozygote-heterozygote base. The cages were observed periodically, and the parameters shown in column 1 were recorded. Only the bodies seen dead were counted as dead mice in litter size determination.

^†^About 30% of the females died during delivery for both models.

^§^The percentage was compared to the theoretical number 100%. *P *< 0.0001 by binomial test.

In further study, we confirmed the significant decrease in survival rate, both in the conventional model and in the Cre-loxP model (Table 3). Also, given that the survival rate of the Cre-loxP model was slightly higher (Table 4A), this further study showed the survival rate dropped from 89% to 56%, which was similar to the conventional knockout model, only 16 pups delivered by 13 breeding pairs in 5 months. We hence inferred that the reduction in survival rate of the offspring resulted from knockout of the Lc3 synthase gene *B3gnt5*.

**Table 4:** Survival rate and reproductive effects of Lc3 synthase knockout

**A. **Heterozygote breeding analysis in Lc3 synthase knockout mice*			
**Outcome of Breeding**	**Conventional Knockout**	**Cre-loxP Knockout**	

Number of delivered offspring	230	249	
Number of surviving mice (survival rate)	135 (59%)^†^	221 (89%)^†^	
Number of breeding pairs	18	5	
Breeding time (months)	6	5.8	
Number of litters	42	33	
Average litter #/breeding pair	2.3	6.6	
Average # of pups/litter (av # surviving/litter)	4.7 (3)	7.5 (6.7)	

**B**. Homozygote breeding analysis of Lc3 synthase knockout mice			

**Outcome of Breeding**	**Conventional Knockout**	**Cre-loxP Knockout**	

Number of offspring	33	21	
Number surviving (survival rate)	3 (9.0%)	12 (57%)	
Number of breeding pairs	6	4	
Breeding time (months)	5	5	
Number pregnant**	9	7	
Average # pregnancies/breeding pair	1.5	1.75	
Average #pups/pregnancy (av # surviving/pregnancy)	3.7 (0.3)	3 (1.7)	

**C**. Embryo experiments on Lc3 synthase knockout mice			

**Group**	**Number of Breeding Pairs**	**Number Pregnant**	**Average Number of Fetuses per Pregnancy**^††^

Conventional	26	13	6.78 ± 2.42
Cre-loxP	3	2	7.00 ± 0.00

*The breeding was carried out with a heterozygote-heterozygote base. The cages were observed periodically, and the parameters shown in column 1 were recorded. Only the bodies seen dead were counted as dead mice in litter size determination.

^†^The percentage was compared to the theoretical number 100%. *P *< 0.0001 by binomial test.

**Pregnancy was indicated by a positive plug and a big belly, but the litter size is sometimes zero here.

^††^At days E15 to E18, fetuses were obtained through dissection of the dam as described in Materials and Methods.

#### Reproductive defects

To compensate for the reduced survival rates, we increased the breeding pairs in order to obtain enough pups for further investigation. As a result, in 6 months, 18 breeding pairs of Lc3 synthase heterozygous knockout mice produced a total of 42 litters, with an average of 2.3 litters per breeding pair, and an average of 3 surviving pups in each litter (Table 4A). These results demonstrated a small litter size and lower pregnancy rate than the average theoretical numbers for mouse breeding. Further, in these 18 breeding pairs, 6 pairs lost fertility after the first litter, which indicated a short reproductive span. Moreover, further breeding showed deeper defects in both Lc3 synthase knockout models, with average of 1 litter per breeding pair in 5 months (1.4 for conventional knockout [19 breeding pairs]; 1.3 for the Cre-loxP knockout method [13 breeding pairs]). Also, the average surviving litter size further decreased to 1.6 pups per litter (26 litters) for the conventional model and 0.9 pups per litter (10 litters) for the Cre-loxP system (Table 3). In addition, about 30% of the dams died during delivery.

Because all of the breeding described in Tables 3 and 4A was carried out with the heterozygote base, we next examined whether homozygotes could mate and produce progeny. According to Mendel's law, homozygote breeding could produce only homozygote offspring. Our genotype results (Table 4B) were consistent with this conclusion. However, the surviving litter size continued to decrease (an average of 0.3-1.7 pups per pregnancy, n = 16 for the two models combined). For the conventional knockout group, nearly no pups survived any delivery (average = 0.3, n = 9). Although the Cre-loxP model produced a few more live pups per pregnancy (average = 1.7, n = 7), we still concluded that, compared to the Lc3 synthase wild-type mice, the heterozygotes and homozygotes (Table 4A and 4B) from both Lc3 synthase knockout models showed severe reproductive defects.

We noted that most offspring died immediately after birth or during delivery because of the death of dams, in either heterozygote or homozygote breeding. To examine the possibility of fetus death in utero, we also performed embryonic studies. The exact date of pregnancy was judged by positive plug formation. At days E15 to E18, the pregnant females were dissected, and the embryos were counted. We found that the total fetus number per pregnancy was within normal range, with an average 6.8 to 7 fetuses per pregnant female (Table 4C). Although we did find 1 to 3 dead fetus bodies in each of the 5 dams in a total of 29 breeding pairs, we assumed that the death of the fetuses was not caused by embryonic lethality. Thus, our embryo experiments shown in Table 4C led us to conclude that Lc3 synthase knockout mice were fertile and that the major reasons for reproductive defects had to do with difficulties in delivery or reduced neonatal viability, the same reasons that ultimately contributed to the overall reduced neonatal survival rate. We could not find a good explanation for the short reproductive span and low pregnancy rate.

#### Fur defects

**Figure 6:**
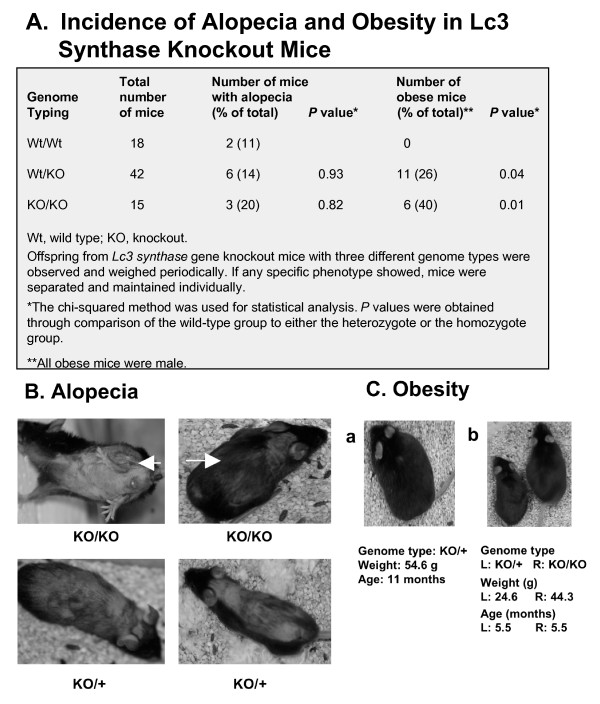
**Alopecia and obesity in Lc3 synthase knockout mice**. **A**. Incidence of alopecia and obesity. A weight of >40 g in a male was counted as obesity. **B**. Alopecia. Fur loss was seen in all three genome types (wild-type data not shown) and could occur anywhere on the mouse's body. The arrowhead indicates where the fur loss caused difficulty in extension of hind limbs, and the arrows show fur growing again with less density. **C**. Late-stage obesity. Mice were weighed periodically. In panel b, the 24.6-g control with a genotype +/- was from the same litter as the obese mouse.

We observed alopecia, or fur loss, in both Lc3 synthase knockout models that affected all regions of the mouse's body (Figure 6A and 6B). The genome typing of these alopecic mice indicated that the lesions occurred in all three genotypes, and the associated *P *values indicated that the differences in results for the three groups were not significant (Figure 6A). Because of the fur loss, some mice acquired skin inflammation, which may have been caused by scabbing. In addition, the fibrous tissues that formed during the healing of these wounds resulted in contractures that prevented mice from fully extending their legs and necks. We sent mice with inflammation to a veterinary diagnostic laboratory at Duke University to test the possible causes. All pathogens for which the mice were tested, including bacteria and viruses, were negative. Generally speaking, the alopecia did not affect the life span of mice, and some of them grew fur again, albeit with less density. However, some of these mice, if the inflammation affected movement, died earlier than their littermates.

#### Late-stage obesity in male knockout mice

To our surprise, in some male homozygous and heterozygous mutant mice, we observed an age-related obese phenotype at about 5 months, these obese mice weighing almost twice as much as their littermates (Figure 6C). We did not observe the same phenomenon in female mutant mice, even at the age of 2 years. About 26% of the heterozygous males and 40% of the homozygous males developed the obese phenotype (Figure 6A) (*P *< 0.05 and *P *= 0.01, respectively), which indicated that the Lc3 synthase gene *B3gnt5 *probably was one of factors that controlled for lack of obesity. Since not all of the null mice developed obesity, we presumed that either Lc3 synthase was not the only factor causing the obese phenotype or the deletion of the Lc3 synthase gene made mice more sensitive to developing obesity. In general, obese mice survived a normal life span of about 2 years. We noted that an increasing number of mutant mice died in 5-15 months, in contrast to the life span of the wild-type littermates. Gross morphological examination revealed splenomegaly and enlarged lymph nodes as major pathological changes.

### B-cell abnormalities of Lc3 synthase knockout mice

Our results from RT-PCR analysis and the ganglioside assay (Figure 4B and Table 1), in agreement with the literature [23], demonstrated that Lc3 synthase was expressed mainly in the developing embryo of wild-type mice, and it could not be detected in embryos at the same stage from homozygous knockout mice. Postnatally, the expression of Lc3 synthase was restricted to wild-type spleen and brain, with particularly high expression in spleen (Figure 4B). As expected, Lc3 synthase expression was completely knocked out in *B3gnt5 *homozygous knockout mice (Figure 4B and Table 1). We compared the mouse brain histology from the three genotypes of Lc3 synthase, KO/KO, Wt/KO, and Wt/Wt, in an extensive side-by-side evaluation of hematoxylin & eosin (H&E)-stained, frozen-section, whole-mount slides of brain, which were cut in the horizontal plane to include cerebellum, hippocampus, basal ganglia, cerebrum, and optic neuron. This analysis failed to reveal any abnormalities of neuronal migration or white matter volume (results not shown). We also did an immunohistochemical analysis using anti-calbindin antibody, and the anti-calbindin results demonstrated no significant differences in the three genotypes--the KO homozygote, the Wt/KO heterozygote, or the Wt homozygote--for immunoreactivity in cerebellum, cortex, or spinal cord (results not shown).

**Figure 7:**
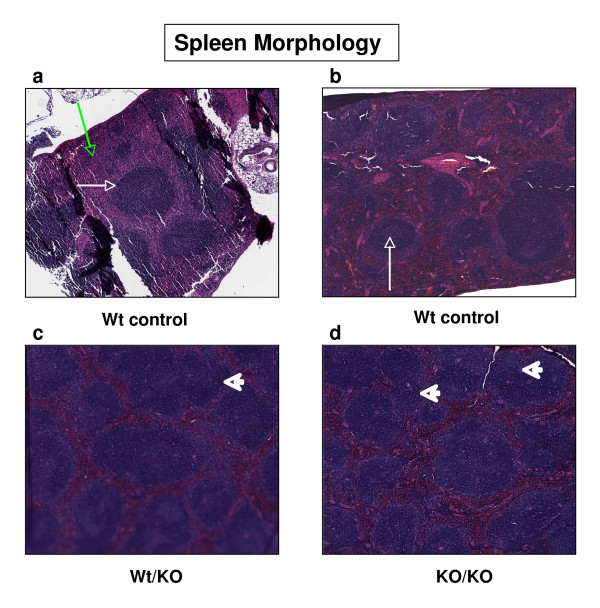
**Spleen morphology of Lc3 synthase-knockout mice**. Mouse spleens from three different genome types of similar age were sectioned and stained. The wild-type controls included a littermate (panel a) and a mouse from a non-knockout colony (panel b). White pulp is indicated by a white arrow in panel a, and the green arrow indicates red pulp. A germinal center is indicated by a white arrow in panel b. In both Wt/KO and KO/KO genotypes (panels c and d) tissue, the arrowhead indicates fused white pulp. The germinal center in the KO/KO mouse spleen (panel d) is not clearly defined.

The preferential expression of Lc3 synthase in spleen prompted us to study its specific role in this organ. The high expression of Lc3 synthase in wild-type spleen and the evidence of splenomegaly and enlarged lymph nodes in *B3gnt5*^KO/KO ^mice prompted us to further study its specific role in cellular regulation. The overall structure of wild-type spleen, with H&E staining, showed normal distribution of splenic nodules (white pulp), germinal center, and red pulp. However, in the heterozygous and homozygous *B3gnt5 *knockout spleens, the marginal zone disappeared, and the limits between the disorganized germinal center and the red pulp were blurred. We also observed a greater amount of white pulp in homozygotes, with some fused to each other, which resulted in enlarged white pulp. In addition, for some splenic nodules, a germinal center architecture disturbance or a missing germinal center was found in *B3gnt5*^KO/KO^mice (Figure 7).

**Figure 8:**
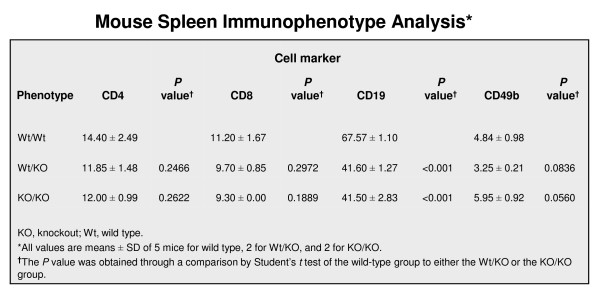
**Spleen immunophenotyping of Lc3 synthase-knockout mice**. Mouse spleen immunophenotype analysis showing the fluorescence intensity of the staining of four antibodies: The intensity from T-cell antibodies (CD4 and CD8) was normal in the Wt/KO or KO/KO phenotype as compared to that in the Wt/Wt phenotype; that for NK cell antibody (CD49b) in the Wt/KO or KO/KO phenotype was similar to that of the Wt/Wt phenotype, with *P *≥ 0.05; and that for B cells (CD19) in the Wt/KO or KO/KO phenotype was reduced by about 39% when compared to that of the Wt/Wt phenotype, with *P *< 0.001.

Because of the histological changes in the spleen of knockout mice, we examined cell classification in knockout spleens and in wild-type spleens by determining the B-cell and T-cell populations in the three different genotypes. For this study, we used flow cytometric analysis with cell-type-specific antibodies. As shown in Figure 7, the B-cell number detected by the anti-CD19 antibody was reduced significantly, by 39% (67.6 vs. 41.5, *P *< 0.001), in homozygous and in heterozygous *B3gnt5 *knockout spleens, when compared to the B-cell number of the wild-type spleen. In contrast, we found that the population of T cells detected by anti-CD4 or anti-CD8 antibodies and the population of NK cells detected by the anti-CD49b antibody were not significantly affected by this Lc3 synthase gene knockout (Figure 8).

### Immunization of *B3gnt5 *Knockout Mice with 3'-isoLM1 or 3',6'-isoLD1

**Figure 9:**
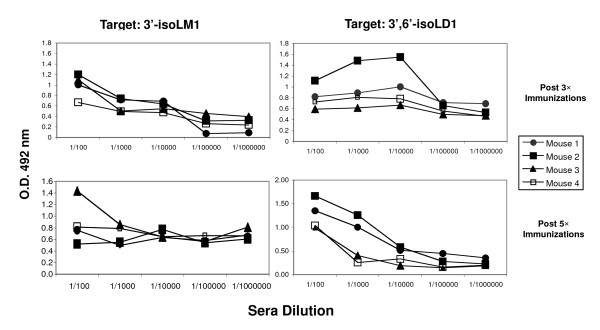
**Anti-IgG response against 3'-isoLM1 and 3',6'-isoLD1**. Four *B3gnt5*knockout mice, each shown as a separate curve, were immunized with purified gangliosides 3'-isoLM1 and 3',6'-isoLD1 that had been conjugated to *Salmonella minnesota *as a carrier and adjuvant. Sera from the immunized mice were tested by ELISA for reactivity against the purified gangliosides (3'-isoLM1 and 3',6'-isoLD1) as optical density readings at 492 nm (OD 492 nm).

Our original purpose in developing Lc3 synthase knockout mice was to create an immunologically naïve host for ganglioside 3'-isoLM1 and/or 3',6'-isoLD1 immunization, so that high-affinity IgG antibodies could be produced. Two hypotheses may be stated as possible explanations for the ability of these mice to produce high-affinity IgG to gangliosides: (1) The absence of synthesized endogenous complex gangliosides makes the mice immunologically naïve, or (2) the lack of complex gangliosides affects immune regulation such that these knockout mice produce IgG instead of IgM antibodies. The *B3gnt5 *homozygous knockout mice were immunized with purified gangliosides, either 3'-isoLM1 or 3',6'-isoLD1, conjugated to *Salmonella minnesota *as a carrier and adjuvant. Mice were minimally immunized s.c. 5 times with the ganglioside conjugates, and sera from the immunized mice were tested by ELISA for reactivity against the purified gangliosides. We have observed positive antibody responses of the IgG type in the immunized mice, and pre-immunized mouse serum was used as the control baseline (data not shown). For both types of ganglioside immunization, midpoint titers from some of the sera (post 3× and post 5× immunizations) are near the 1:10,000 titer range, which is appropriate for progressing to hybridoma generation (Figure 9). It should also be noted that these are IgG subclass titers. In virtually all past attempts, including a variety of different immunization schemes and numerous strains of immune competent mice (e.g., C57BL/6, CH3, and SJL/j), to generate ganglioside-reactive hybridomas, only IgM antibodies were generated. Nevertheless, the use of the immunologically naïve knockout mice has enabled us to successfully induce IgG responses to 3'-isoLM1 and 3',6'-isoLD1 in this immunization strategy. However, in the course of evaluating them by making a hybridoma fusion of spleen cells with myeloma cells, we found that although the fused hybridoma cells could survive in hypoxanthine aminopterin thymidine (HAT) medium, very few positive clones were identified, and these proliferated with low efficiency (data not shown).

## Discussion

The mouse Lc3 synthase gene *B3gnt5 *was first cloned by Henion et al. in 2001, whose work indicated that the key function of the Lc3 synthase enzyme is the regulation of lacto-neolacto-series ganglioside synthesis during embryonic development and brain morphogenesis [23]. Postnatally, the expression was restricted to splenic B cells, the placenta, and cerebellar Purkinje cells, where it co-localized with HNK-1 reactivity [23]. We sought to further elucidate the role of this enzyme.

In the present study, we created two genetic models by disrupting the *B3gnt5 *gene, and thus the expression of Lc3 synthase. We used two different strategies to establish these knockout mice and then compared the phenotypic changes in these two models. Both models showed that lack of Lc3 synthase resulted in complete deficits in downstream gangliosides of the lacto-neolacto cascade. Nevertheless, mice with totally deficient lacto-neolacto-series gangliosides experienced normal embryonic development and were born at the expected Mendelian ratio, and if they survived delivery, could be kept viable postnatally. However, about 9% of all offspring displayed a dwarf phenotype, and 11% died in early life, and genome typing results confirmed that the Lc3 synthase-null mice tended to die early.

Mice carrying the homozygous deletion of Lc3 synthase were viable. Breeding of homozygous mutant mice produced live pups, albeit with reduced survival rate, which may indicate reproductive defects. Recently, Biellmann et al. reported the disruption of the *B3gnt5 *gene in mice, and breeding of heterozygous mice failed to produce any viable homozygous progeny [24]. They believe that the *B3gnt5 *gene is essential to pre-implantation development of the murine embryo. The reason for the discrepancy with our results is not clear. We characterized our knockout mice by Southern blot analysis, RT-PCR, and PCR, and by sequencing genomic DNA clones, confirming that exon 4 of Lc3 synthase of homozygous mice was deleted. Most recently, Kondo et al. from Furukawa's group also reported *B3gnt5*-deficent mice (*B3gnt5*-/-) were viable, showing by sensitivity analysis that GSLs are not pivotal receptors for Subtilase cytotoxin *in vivo *with such mutant mice lacking lacto/neo-lacto series GSLs [25].

As our mutant mice matured, pleiotropic phenotypic changes occurred, including dwarfism (Figure 5), fur loss (Figure 6B), and obesity (Figure 6C). A vexing issue for either dwarfism or fur loss was the lack of a satisfactory explanation for these phenotype changes, which occurred not only in mutant mice, but also in the wild-type littermates. The fur loss could not be ascribed to fighting between mice, and this phenotypic change has not been reported in similar studies of ganglioside knockout mice. However, a dwarf phenotype in knockout mice has been reported in several studies [19,26,27], such as those with knockout of the gene encoding acetylcholinesterase and *Ugcg*, encoding GlcCer synthase, which catalyzes UDP-activated glucose to ceramide. Those genes knocked out were totally different from the *B3gnt5 *gene we knocked out here. The systemic disruption of *Ugcg *in mice is lethal during early embryogenesis, and the offspring with this specific gene deficiency in brain gained less weight than their controls in the first 16 postnatal days. Because the deaths of all homozygous mice that occurred within 24 days in those studies were associated with abnormalities in neural cell differentiation, it is difficult to draw any conclusions with regard to the dwarf phenotype from our study.

Of interest in our study was that in the wild-type mice that were not littermates of a knockout group we could not identify any similar phenotypic changes. These data led us to conclude that the dwarfism in wild-type littermates, differing from the non-knockout control colony, may have been caused by some genetic background differences, occurring in germ line transmission, or may have resulted from knockout techniques or recombination processes. Recently, Reed and colleagues compiled body-weight information on about 2000 viable knockout mice, and they reported about 31% of the knockout mice weighed less than controls. They assumed that more than 6000 genes could contribute to mouse body weight [26]. Thus, it is worth emphasizing that Lc3 synthase may be involved in some way during development or in the maintenance of body weight.

As for defects in reproductive capability, considerable data has been accumulated to date about the biological effects of gangliosides on fertility. Male mice in which cerebroside sulfotransferase (CST, EC 2.8.2.11) has been knocked out lose fertility because of a block in spermatogenesis, while females are able to breed [28]. Similar observations have been made for mice in which ceramide galactosyltransferase (CGT, EC 2.4.1.62) [29] and GM2 synthase (*Galgt1*) [30] are knocked out. Our data definitely indicate that the absence of lacto-neolacto-series gangliosides affected the reproductive system, but predominantly in the female mice. Although the lack of Lc3 synthase did not appear to affect the capability for fertilization, after their first pregnancy, at least 33% of the females lost fertility permanently, even though breeding was continuously performed. This would suggest that a short reproductive span results from Lc3 synthase knockout. In our further study, we observed that the average birth rate was only about one litter per breeding pair. Thus we could not exclude the possibility that Lc3 synthase knockout progressively disrupted fertility, despite normal fertilization in the first pregnancy.

We also investigated the effect of lack of Lc3 synthase on the pups' development in utero. We expected that the Lc3 synthase gene would be essential for embryonic development, as indicated by the literature [26]. However, this was not the case. Random heterozygous cross-breeding gave an expected Mendelian inheritance, identified by genome typing (Table 2, Fetus group). The general morphology of newborn pups was normal (data not shown). Thus, Lc3 synthase knockout was not lethal in fetal development. However, delivery was seldom successful, in that about 30% of the dams died during delivery. Dissections after the death of the dam usually revealed 5-7 fully developed, but dead fetuses in the uterus. Moreover, even if the dams overcame the difficulties of delivery, about 60% of the pups died immediately after birth, and about 71% of the dead pups were KO homozygotes (Table 2). The morphology of dead newborn pups displayed no significant abnormalities that might indicate the cause of death.

Our analysis of the phenotypic changes described above, considered in the context of the relevant literature, subsequently led us to investigate the role of lacto-neolacto gangliosides in spleen. Although the existence of clustered gangliosides in the plasma membrane in human lymphocytes has been shown by electron microscopy [31], the functions of these gangliosides have not been delineated completely. Some evidence indicates that they have several regulatory effects on monocytes and myeloid cells [7], as well as T cells of the immune system [32]. It is well known that spleen is a secondary immune organ. We observed enlarged spleen and lymph nodes in dead mice and identified the spleen to be the organ with the major Lc3 synthase expression in adult wild-type mice (Figure 4B). Thus we hypothesized that these changes in the spleen indicated a relationship to the biological roles of gangliosides.

Remarkably, we found that depletion of Lc3 synthase, conceivably abolishing the formation of lacto-neolacto gangliosides, led to a decrease of B-cell numbers in spleen in Lc3 synthase-null mice. Also, the spleen morphology in null mice was changed, with some germinal centers--the major B-cell location--totally gone. At first, because we did not fully understand the significance of these morphological changes, we continued to employ the Lc3 synthase-null mouse as a host for the lacto-neolacto ganglioside immunization according to our experimental design. Using the knockout mouse model, we elicited an IgG response, but with moderate or low titers, and the hybridoma fusion of immunized spleen cells using the conventional polyethylene glycol fusion method had low viability. During the fusion experiments, we observed that the fused cells survived in HAT medium for several months, but did not proceed to proliferation. Thus the underlying mechanism to be inferred from our data is that Lc3 synthase knockout severely affected the functional integrity of B cells.

The expression of Lc3 synthase in splenic B cells is interesting in light of the high level of activity with gangliosides synthesized through GM3 and GM1 [33]. A rare species of hybrid ganglioside structures containing extended lactosamine structures on a GM1 core was identified previously in glycolipid extracts of splenic B cells, but not T cells [33]. These gangliosides were shown to have the core structure GlcNAc(1-3)Gal(1-3)GalNAc-R. It seems likely that Lc3 synthase is involved in the synthesis of this GlcNAc(1-3) linkage. Although the structural confirmation and significance of these findings await further analysis, these results may reveal a previously undescribed role for Lc3 synthase in regulating synthesis of these unique glycolipids. The exact functions of lacto-series glycolipids have remained elusive, with the exception of a few specific processes. The disruption of the Lc3 synthase gene in mice should provide valuable insight into the functional involvement of this glycolipid class in developmental and physiological pathways.

We have not seen any report on obesity in ganglioside knockout mice. The incidence of obesity was about 40% in null mice, and it occurred only in male mutant mice. There are genetic and immunologic data that strongly suggest that type I diabetes may be associated with autoimmune antibodies against gangliosides, mainly disialo- or polysialo-gangliosides [34]. Also, a growing body of evidence indicates that mice without the capacity to synthesize the a-series GM3 ganglioside display an increased insulin sensitivity, and these mutant mice are protected from high-fat-diet-induced insulin resistance [14]. Thus, gangliosides may be involved in occurrence of obesity. In concordance with this hypothesis, Lastres-Becker et al. recently reported the deletion of *SCA2*, coding ataxin-2 protein and causing spinocerebellar ataxin type 2 disorder, and found similar phenotypic changes of obesity and reduced fertility [35]. Interestingly, they also found significant changes in the expression level of gangliosides, mainly GM1, GD1a, GD1b, and GT1b. They suggested that gangliosides are characteristic constituents of membranes that are involved in the insulin-signaling pathway. Although the gene they knocked out was different from ours, the study did indicate an interconnection between phenotypic changes of obesity, reduced fertility, and ganglioside level. Nevertheless, further experiments are necessary to better define the possible relationship of our knockout models to obesity.

Despite significant multiple phenotypic changes observed here in some of the homozygote and heterozygote mice, other mice were able to survive in approximately normal health for more than one year. This variability remains to be investigated, and there are several possible explanations: First, Lc3 synthase, which functions at the beginning of the lacto-neolacto-series pathways, has an impact on the genes that affect the formation of multiple molecules along the whole pathway cascade. Thus, Lc3 synthase knockout may cause multiple phenotypic changes. Second, because surface ganglioside composition differs--possibly individually--in quality and quantity, sensitivity to knockout may differ individually. Third, the extent of ganglioside distribution and functional overlapping or compensation may also vary by individual experimental animal, especially in a knockout model [36]. All of these individual differences may be contributing to phenotypic variations. Furthermore, evidence has already been presented that there are hybrid ganglioside molecules in the lacto-neolacto-series pathways with the structure of *N*-acetylgalactosamine, which has been defined as the major component of the a- and b-series pathways [37], indicating the possibility of functional overlapping within the different ganglioside pathways. In addition, we also observed some subtle neural phenotypic changes occurring in Lc3 synthase-mutant mice, such as hind-limb weakness, tremor, and seizure onset, demonstrating possible CNS damage.

It is known that gangliosides are poor antigens [38], and raising antibodies against gangliosides in wild-type mice is difficult because of the poor antigenicity of the gangliosides and because of immunological tolerance in animals. We previously used 3'-isoLM1 and 3'6'-isoLD1 as targets to immunize several strains of mice and encountered poor humoral immune responses, producing only IgM antibodies [11], which confirmed poor ganglioside antigenicity. On the other hand, in addition to their overexpression on the surface of a wide variety of tumors, gangliosides are involved as autoantigens in the pathophysiology of human autoimmune disorders in the peripheral nervous system and CNS [3,38-41]. Endogenous and exogenous antiganglioside antibodies contribute to induction of human autoimmune nerve pathology. Nevertheless, anti-GD1a IgG antibodies have been generated in *GalNacT *(β-1,4-*N*-acetylgalactosaminyl transferase)-null mice [22,39]. Most recently, we have successfully isolated specific anti-3'-isoLM1 and anti-3',6'-isoLD1 IgG clones from our immunized Lc3 synthase knockout mice, 50% of the clones being IgGs, in an attempt to use a low-toxicity, high-efficiency fusion method via a Sendai virus envelope. Further, after single cell cloning, one of the clones, GMab-1 (IgG_3 _subclass), was established to specifically recognize lacto-series gangliosides with high affinity. Significantly, it was reactive against human glioma samples in immunohistochemistry assays and thus demonstrated potential as a good reagent for diagnosis and therapy [42].

## Conclusions

These novel results from the disruption of the *B3gnt5 *gene in mice demonstrate unequivocally that lacto-neolacto series gangliosides are essential to multiple physiological functions, especially the control of reproductive output, and spleen B-cell abnormality. However, our results suggest the *B3gnt5 *gene is not essential for mouse embryonic development. We also employed the Lc3 synthase knockout model, lacking the lacto-neolacto-series, to successfully generate an IgG response against 3'-isoLM1 and 3',6'-isoLD1 gangliosides. This led to the subsequent first IgG antibody generation, which was recently achieved by our group [42].

## Methods

### Generation of Lc3 synthase gene knockout mice

Two strategies were used to produce Lc3 synthase gene *B3gnt5 *knockout models. For model 1, *B3gnt5 *was knocked out by a conventional method. A 12.3-kb region used to construct the targeting vector was first subcloned from a positively identified BAC clone. The region was designated such that part of intron 3 and all of exon 4 of the gene were deleted and replaced with the MC1NeoPolyA selection cassette. The targeting vector was confirmed by restriction enzyme analysis after each modification step and by DNA sequencing with primers designed to read upstream of intron 3 and downstream of exon 4 and the *neo *gene. This vector was transfected into C57BL/6 ES cells. After homologous recombination, the positive cells carrying a targeted allele were screened by PCR and microinjected into C57BL/6 blastocysts, and *B3gnt5 *knockout mice were established through final germ-line transmission by continuous breeding with the C57BL/6 strain.

For model 2, *B3gnt5 *was disrupted by a Cre-loxP system. Since the coding region of this gene resides only within exon 4, exon 4 of *B3gnt5 *was flanked by a pair of loxP sites. In order to do this, we first designed specific primers to amplify the *B3gnt5 *gene fragments from 129sv strain ES cells. A PCR product was used as a probe to generate *B3gnt5*-positive BAC clones from the mouse 129sv strain by microarray screening. One genomic BAC clone was used to construct the targeting vector containing a 7.9-kb genomic DNA sequence with a floxed Lc3 synthase targeting allele. The resulting targeting clone, pOSfrt-loxP-*B3gnt5*, confirmed by restriction enzyme analysis and DNA sequencing, was transfected into the 129sv ES cells. Eighty clones were screened by Southern blot to identify the recombinant cells carrying a floxed *B3gnt5 *allele, which were then microinjected into C57BL/6 blastocysts to generate the C57BL/6-129sv chimeric mice. Chimeric males were then mated to C57BL/6 females. Germ-line transmission of the floxed *B3gnt5 *allele was established by PCR screening of agouti offspring. Mice bearing the floxed *B3gnt5 *allele were mated to the general Cre-deleter mice (The Jackson Laboratory mice database: B6.FVB-TgN[EIIa-Cre]), which resulted in excision of the floxed *B3gnt5 *sequence.

### Maintenance of knockout mice

All of the mice were kept in the Cancer Center Isolation Facility at Duke. From one to five animals were maintained in an individual cage on a regular dark/light cycle with unrestricted access to food and water. The mice, of three different genome types, were maintained through intercross breeding between heterozygotes from the same generation or through backcross breeding with one of the parents. Knockout of the *B3gnt5 *allele was confirmed by PCR of DNA prepared from tail biopsies collected at weaning. To determine homozygotes of the *B3gnt5 *allele, PCR products were purified and sequenced to ensure their homogeneity.

All breeding was done in a C57BL/6 background. Animals were maintained in accordance with National Institutes of Health guidelines for the care and use of laboratory animals and under the approval of the Duke University Medical Center Institutional Animal Care and Use Committee.

### Genotyping of mice by PCR

A mouse tail kit (Qiagen, Valencia, CA) was used to extract genomic DNA. For the conventional knockout mouse model, genotyping was performed by PCR with primers of 5'exon 4 (5'ATGAGACTGTTTGTTAGC) and 3'exon 4 (5'GATTGTGGAAAGAATCAA). The expected DNA product size for the wild type was 0.5 kb. Also, *neo *gene primers with sequences as follows were used.

Neo1 R (5'GCTACACAAGTGGCCTCTGGCCTCGCA)

Neo7 R (5'CTTCAGGCTATGAAACTGACACAT)

The size of the targeted DNA fragment was 1.9 kb.

For Cre-loxP knockout mice, PCR was performed with the same exon 4 primer set plus Cre primers.

loxP (5'TTTGAGTTTTCTCGAGATAACT)

and

SAR (5'GGTACCAATTGACATAAGGAAATGGGACTTC)

The size of the floxed *B3gnt5 *allele was 4.3 kb; that for the knocked out *B3gnt5 *allele was 2.1 kb. PCR cycling was based on the different primer sets.

### RT-PCR

Total RNA was extracted from frozen liver, spleen, and brain of adult mice or from head and body of day E17 fetuses by using an RNeasy mini isolation kit (Qiagen) as described by the manufacturer. About 5 μl (1 μg) of total RNA was used for synthesis of first-strand cDNA by using SuperScript II reverse transcriptase and a random primer (Invitrogen, Carlsbad, CA). Subsequently, 1 μg of cDNA was used for PCR with the same exon 4 primer set as was used for the above-described genotyping. Mouse β-actin (primers from Invitrogen Biosource) was used as an internal quality control to monitor RNA recovery. Cycling was performed according to standard procedure. Samples were run on a 1% agarose gel.

### Perfusion, dissection, and histological analysis of multiple organs

The anesthetized animals were perfused through a cardiac tube with 4% paraformaldehyde in phosphate buffer for approximately 2 h. Organs from the three mouse genotypes, including heart, lung, spleen, cortex, cerebellum, kidney, pancreas, stomach, small intestine, bladder, uterus, testes, and skeletal muscle, were collected and fixed in 4% paraformaldehyde for 12 h. The fixed tissues were either protected in 30% sucrose for 2 days at 4°C, then flash-frozen in Tissue-Tek OCT compound (Sakura Finetek USA Inc., Torrance, CA), or directly paraffin embedded. Frozen or paraffin-embedded sections (5-10 μM) were cut and stained with H&E by using standard techniques.

### Behavioral analysis of knockout offspring

The knockout offspring from heterozygous breeding were matched either with wild types or with littermates for behavioral studies, including appearance, weight, survival rate, eating and drinking, and mating. Once any special phenotype appeared, the mice were placed and observed separately and then compared to their littermates for at least 3 months.

### Evaluation of reproductive performance

Heterozygous or homozygous knockout mice of similar ages were bred from 5 to 9 months to assess the fertility of mutants. Pregnancy was confirmed by observation of positive plugs. The day when a plug appeared was counted as day 0.5. The number of litters during the breeding process, the litter sizes, and the number of surviving pups and dead pups were recorded. For fetal studies, pregnant females were dissected at days E15 to E18 while anesthetized. The fetuses per pregnancy were counted and totals recorded. Fetuses were genotyped by using the same mouse tail kit as was used for adult mice.

### Ganglioside assay

The organs were removed by dissection from anesthetized mice of the three different genotypes. Also, fetal tissues were taken from the anesthetized dam, quickly frozen in dry ice, and processed for the ganglioside assay. The total ganglioside fraction was isolated essentially as previously described [37]. Quantitative determination was made of lipid-bound sialic acid (total gangliosides) by the resorcinol procedure, and the distribution of the major ganglioside species was determined by densitometric evaluation of a high-performance thin layer chromatography (TLC) chromatogram after resorcinol visualization [43]. The lactoseries gangliosides 3'-LM1, 3'-isoLM1, and 3',6'-isoLD1 were quantitated by TLC-immunostaining using the specific monoclonal antibodies LM1, TR4, and DMab 22, respectively [37].

### Spleen immunophenotyping by fluorescence-activated cell sorting

Flow cytometric analyses were performed on spleen cells by following a standard protocol. Briefly, harvested cells were washed twice with ice-cold phosphate-buffered saline (PBS), and then, cell-surface nonspecific antibody binding was blocked with 10% normal goat serum in PBS (Sigma, St. Louis, MO). Blocked cells were mixed with 10 μg/ml of either anti-CD4 (clone L3T4), CD8 (53-6.7), CD19 (1D3), CD49b (DX5), or the appropriate isotype-specific controls in ice-cold PBS-1% BSA (Invitrogen). All antibody fluorescein conjugates were acquired from BD Pharmingen (San Jose, CA). The cells were incubated by rotating them in the dark for 60 min at 4°C, washed in PBS-1% BSA, and then resuspended in 0.05% paraformaldehyde/PBS for acquisition on a FACSCalibur flow cytometer (BD Biosciences, San Jose, CA). The data were analyzed with BD CellQuest Pro software (BD Biosciences) and represented as median fluorescence intensity as compared to the appropriate isotype control antibody.

### Immunization of homozygous mice with gangliosides and testing by ELISA

Homozygous knockout mice, age 2-4 months, were used for immunization. Purified gangliosides, either 3'-isoLM1 or 3'6'-isoLD1, conjugated with *Salmonella minnesota *were used as antigens. The conjugates were prepared as follows. Gangliosides were dissolved in ethanol and acid washed. Salmonella particles in water were added in the weight ratio ganglioside-Salmonella particles 1:10. After incubation for 1 h at +40°C, the conjugated particles were diluted with PBS and stored frozen until use. Then, 5 μg of each antigen was injected subcutaneously 3-5 times at intervals of about 20 days. Sera were collected and tested by ELISA for reactivity against the gangliosides as optical density readings at 492 nm. Pre-immunized mouse serum was used as the control baseline.

### Statistical Analysis

Data were expressed as mean ± standard deviation. Results having a *P *value of < 0.05, or a *P *value of < 0.01 by the Student *t *test, chi square test, or binomial test, were considered to be significant.

## Abbreviations

CGT: ceramide galactosyltransferase; CNS: central nervous system; CST: cerebroside sulfotransferase; ES: embryonic stem; H&E: hematoxylin and eosin; KO: knockout; PBS: phosphate-buffered saline; PCR: polymerase chain reaction; RT-PCR: reverse transcription polymerase chain reaction; WT: wild type.

## Competing interests

The authors declare that they have no competing interests.

## Authors' contributions

CK initiated and directed the study, participated in the design of the study, and made substantial revisions to the manuscript. JC participated in the design of the study, drafted and wrote several revisions of the manuscript, constructed the *B3gnt5 *targeting vector, screened ES cells for homologous recombination, and was responsible for mouse breeding, phenotypic characterization, and statistical analysis of all data. JEM and PF extracted and analyzed GSL profiles in knockout mice and made significant revisions to the manuscript. JL and CP performed the embryo isolation, mouse dissection, and slide fixation. RM prepared the H&E slides and contributed the critical interpretation of the histology. DB initiated and directed the study and made significant revisions to the manuscript. All authors read and approved the final manuscript.
